# Reassessment of SST4 Somatostatin Receptor Expression Using SST4-eGFP Knockin Mice and the Novel Rabbit Monoclonal Anti-Human SST4 Antibody 7H49L61

**DOI:** 10.3390/ijms222312981

**Published:** 2021-11-30

**Authors:** Amelie Lupp, Blanca Ehms, Ralf Stumm, Johannes Göckeritz, Christian Mawrin, Stefan Schulz

**Affiliations:** 1 Institute of Pharmacology and Toxicology, Jena University Hospital, D-07747 Jena, Germany; blanca.ehms@gmail.com (B.E.); Ralf.Stumm@med.uni-jena.de (R.S.); goeckeritz.johannes@web.de (J.G.); Stefan.Schulz@med.uni-jena.de (S.S.); 2 Institute of Neuropathology, University Hospital, Otto-von-Guericke-University, 39120 Magdeburg, Germany; christian.mawrin@med.ovgu.de

**Keywords:** somatostatin, somatostatin receptor, SST4, antibody, immunohistochemistry, tumors

## Abstract

Among the five somatostatin receptors (SST1–SST5), SST4 is the least characterized, which is in part due to the lack of specific monoclonal antibodies. We generated a knockin mouse model that expresses a carboxyl-terminal SST4-eGFP fusion protein. In addition, we extensively characterized the novel rabbit monoclonal anti-human SST4 antibody 7H49L61 using transfected cells and receptor-expressing tissues. 7H49L61 was then subjected to immunohistochemical staining of a series of formalin-fixed, paraffin-embedded normal and neoplastic human tissues. Characterization of SST4-eGFP mice revealed prominent SST4 expression in cortical pyramidal cells and trigeminal ganglion cells. In the human cortex, 7H49L61 disclosed a virtually identical staining pattern. Specificity of 7H49L61 was demonstrated by detection of a broad band migrating at 50–60 kDa in immunoblots. Tissue immunostaining was abolished by preadsorption of 7H49L61 with its immunizing peptide. In the subsequent immunohistochemical study, 7H49L61 yielded a predominant plasma membrane staining in adrenal cortex, exocrine pancreas, and placenta. SST4 was also found in glioblastomas, parathyroid adenomas, gastric and pancreatic adenocarcinomas, pheochromocytomas, and lymphomas. Altogether, we provide the first unequivocal localization of SST4 in normal and neoplastic human tissues. The monoclonal antibody 7H49L61 may also prove of great value for identifying SST4-expressing tumors during routine histopathological examinations.

## 1. Introduction

Somatostatin is a cyclic neuropeptide that regulates the release of various neurotransmitters in the central and peripheral nervous system and the secretion of a large number of hormones from the anterior pituitary, pancreas, and endocrine cells within the gastrointestinal tract. Additionally, it causes a reduction of gastrointestinal tract motility and gallbladder contractility. Finally, somatostatin exerts antiproliferative effects and plays a regulatory role in the immune system [1]. The biological functions of somatostatin are mediated via a family of five G-protein-coupled receptors, termed SST1 to SST5, that are expressed in varying patterns and density throughout the body, including brain, pituitary gland, neuroendocrine cells of the bronchopulmonary and gastrointestinal tract, pancreatic islets, adrenal glands, and the immune system. Somatostatin receptors have also been found in pituitary adenomas and in neuroendocrine tumors of different origins where they mediate inhibitory effects on both hormone secretion and tumor growth. Apart from the tissue distribution, SSTs also differ in their affinity for synthetic somatostatin analogs, intracellular signaling pathways, and biological functions [1].

Little is known about SST4 compared to other SSTs, for which highly specific monoclonal antibodies have been available for years and which, as a result, could be examined more closely in terms of their occurrence in tissues and their mode of action. The existing knowledge is based on mRNA expression analyses, receptor autoradiography studies, immunostaining with polyclonal antibodies, SST4 knockout mouse models, and SST4-transfected cell lines [1,2].

Regarding signaling pathways, it is known that SST4, by coupling to G_i/o_ proteins, inhibits adenylate cyclase, thus leading to reduced cAMP production [3]. In rat cortical neurons and retinal and dorsal root ganglion cells, SST4 reduces intracellular Ca^2+^ concentrations by inhibiting voltage-dependent Ca^2+^ channels and activates inwardly rectifying potassium (GIRK) and M channels. This results in membrane hyperpolarization and a subsequent reduction of Ca^2+^ and Na^+^ influx through voltage-dependent Ca^2+^ and TRPV1 channels [3,4,5,6,7,8,9,10]. SST4 seems to be the only family member that not only inhibits cell proliferation via complex regulatory mechanisms, such as tyrosine phosphatase SHP-2 activation [11] and upregulation of the cyclin-dependent kinase inhibitor p21 [12], but also stimulates cell proliferation via an activation signal to protein kinase C and by MAP kinase-mediated serine phosphorylation leading to activation of STAT3 [3].

With respect to SST4 trafficking, observations of an instant dissociation of receptor and ligand after ligand binding, subsequent internalization, and rapid recycling in cells transfected with the human SST4 [13] contrast with those that could not find any internalization of the receptor in rat tissue or in cells transfected with rat SST4 [14,15]. Therefore, species-specific differences in behavior and possibly also in signaling mechanisms of SST4 are to be assumed [1].

Concerning function, SST4 knockout mice have a higher number of spontaneous epileptic seizures than controls, suggesting an important role for SST4 in the hippocampus [4], consistent with the observation of reduced somatostatin binding in the hippocampal CA1 area in these mice [2]. SST4 may also be important in memory, cognition, and learning performance and has an impact on behavior in stressful situations [16,17,18]. Because it protects against inflammation and hyperalgesia, SST4 is being evaluated as a therapeutic target for a novel class of anti-inflammatory and/or analgesic drugs [9,19,20,21,22,23]. There is also evidence that SST4 may be a pharmacological target for Alzheimer’s disease [24,25].

Using mRNA analysis and polyclonal antibodies, SST4 has been previously localized in rats in diverse brain areas, such as in layers I–VI of the neocortex, the hippocampus formation, the hilar region of the dentate gyrus, the amygdala, the hypothalamus, the striatum, the nucleus accumbens, the globus pallidus, the olfactory bulb, and other structures of the olfactory system [15,26,27,28]. It has also been shown to be expressed in retinal ganglion cells [6] and in dorsal root ganglia [22,29]. Compared to the CNS, the presence of SST4 in the peripheral organs in rats is less well characterized. Here, SST4 mRNA and protein expression have been found in the lung, heart, and placenta [26,30]. Using polyclonal antibodies, SST4 receptors have been discovered in the human cortex, hippocampus, nucleus ruber, globus pallidus, cerebellum, and medulla [31], as well as in the exocrine pancreas [32,33], the parathyroid glands, the bronchial glands, the fundic glands of the stomach, the Brunner’s glands in the duodenum, and the distal tubules of the kidney [32]. Additionally, very recently, in a SST4 humanized mouse line, SST4 expression has been disclosed in the cerebral cortex, the olfactory bulb, the hippocampus, the amygdala, and in the trigeminal ganglia [34].

In human tumors, SST4 expression was often recorded along with other SST family members [35,36,37,38,39,40,41,42,43,44,45,46,47,48,49,50,51,52,53,54,55]. However, the specificity of the polyclonal antibodies used for SST4 staining was also questioned by some of the authors (e.g., [45,48,51]). Most of these studies revealed that SST4 was either not expressed in the tumors investigated or at least present to a significantly lower degree compared to the other SSTs. In all cases, only cytoplasmic staining of the cells was observed.

To address the lack of suitable specific monoclonal anti-SST4 antibodies for the localization of SST4 in mouse or human tissues, we generated an SST4-eGFP knockin mouse line and a novel rabbit monoclonal anti-human SST4 antibody as novel tools to facilitate unequivocal SST4 receptor localization. Using anti-GFP antibodies and mRNA in situ hybridization, we show that the novel mouse line enables a detailed localization of SST4 in different mouse tissues. We also demonstrate that the novel anti-human SST4 antibody 7H49L61 is well suited both for immunoblot analysis in basic research and for immunohistochemical staining of routine clinical pathology samples. This antibody was then used for the evaluation of SST4 expression in a large set of formalin-fixed paraffin-embedded human normal and neoplastic tissue samples in order to obtain a broad expression profile both for the human body and for human tumors.

## 2. Results

### 2.1. SST4-eGFP Expression in the Knockin Mouse Line

**Figure 1:**
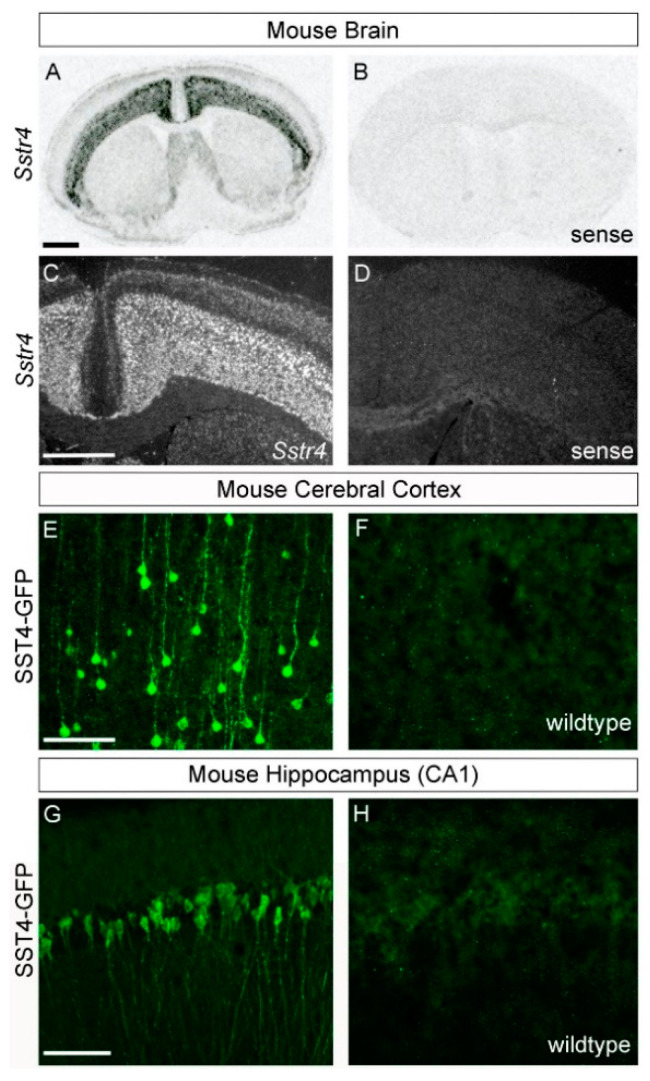
*Sstr4* expression in the mouse brain. (**A**,**B**) X-ray autoradiograms show coronal brain sections after in situ hybridization (ISH) with ^35^S-labeled *Sstr4* antisense (**A**) and *Sstr4* sense riboprobes (**B**). (**C**,**D**) Darkfield micrographs show the cerebral cortex in emulsion-dipped sections after ISH with ^35^S-labeled *Sstr4* antisense (**C**) and *Sstr4* sense riboprobes (**D**). (**E**–**H**) Confocal images show the cerebral cortex (**E**,**F**) and hippocampal subregion CA1 (**G**,**H**) of SST4-GFP (**E**,**G**) and wild-type mice (**F**,**H**) after anti-GFP immunostaining. Scale bars: (**A**,**C**) 1 mm; (**E**,**G**) 100 µm.

**Figure 2:**
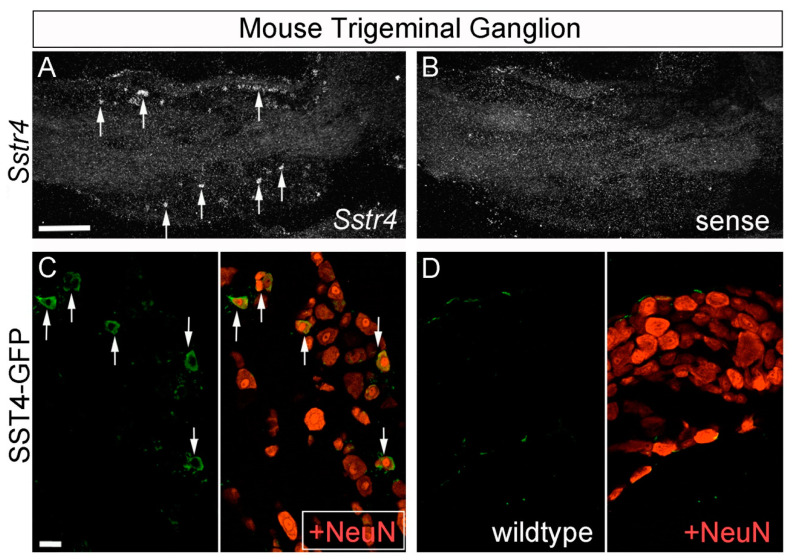
*Sstr4* expression in the mouse trigeminal ganglion. (**A**,**B**) Darkfield micrographs show emulsion-dipped sections of the trigeminal ganglion after in situ hybridization with ^35^S-labeled *Sstr4* antisense (**A**) and *Sstr4* sense riboprobes (**B**). Arrows in A identify *Sstr4*-positive neurons. (**C**,**D**) Confocal images show double immunofluorescence stainings with antibodies for eGFP (**green**) and NeuN (**red**) in trigeminal ganglia of SST4-eGFP knockin (**C**) and wild-type mice (**D**). Arrows in (**C**) identify SST4-eGFP-positive NeuN-positive neurons. Scale bars: (**A**) 500 µm; (**C**) 25 µm.

In C57BL/6-wild-type mice, in situ hybridization experiments revealed high levels of *Sstr4* mRNA expression in the infragranular cortical layers but very low levels in the supragranular cortical layers (Figure 1A,C). *Sstr4*-positive neurons were also observed in trigeminal ganglia (Figure 2A). Similarly, eGFP fluorescence immunostaining in SST4-eGFP knockin mice revealed strong SST4 expression in cortical pyramidal cells and in the pyramidal cells of the hippocampal areas CA1–CA3 (Figure 1E,G). In the granule cells of the dentate gyrus, in contrast, only low SST4 expression was noticed. In trigeminal ganglia, mainly small- to medium-diameter neurons were SST4 positive (Figure 2C). No immunostaining was observed in wild-type mice, which lack SST4-eGFP expression (Figure 1F,H and Figure 2D).

**Figure 3:**
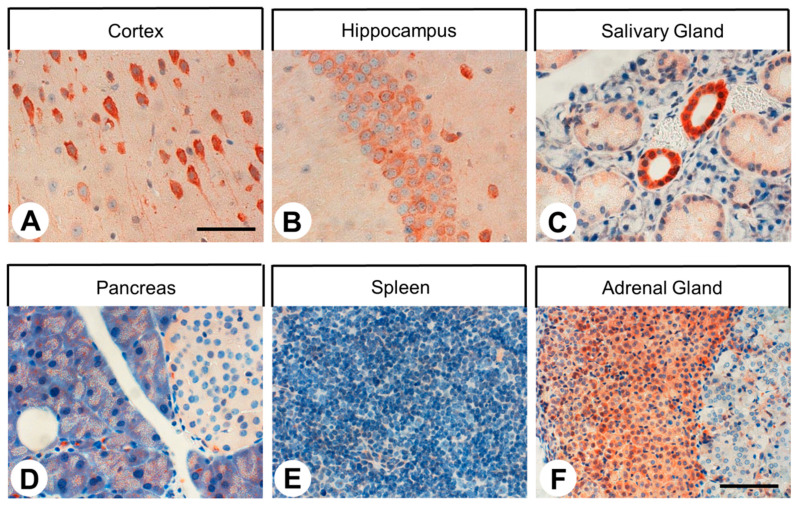
Evidence of SST4 expression in SST4-eGFP knockin mice. SST4 was detected immunohistochemically in tissue sections from SST4-eGFP knockin mice using a commercial rabbit anti-eGFP antibody (ab290, Abcam, Cambridge, UK; 1:1000). Scale bar: (**A**–**E**) = 60 µm; (**F**) = 100 µm.

In the subsequent eGFP stainings of paraffin sections of different tissues from SST4-eGFP knockin mice, a strong immunosignal was noted in the pyramidal cells of the cortex and of the hippocampal areas CA1–CA3, in neurons of the amygdala, in fibers of the posterior pituitary, in excretory ducts of the salivary glands and the pancreas, and in the adrenal cortex. A slight staining was also observed in some cell populations of the anterior pituitary, in the acinar cells of the exocrine pancreas, and in the distal tubules of the kidney. In contrast, no noticeable SST4 expression could be detected in the lung, liver, pancreatic islets, adrenal medulla, thymus, spleen, or lymph nodes (Figure 3).

### 2.2. Specificity of the Monoclonal Rabbit Anti-Human SST4 Antibody 7H49L61

**Figure 4:**
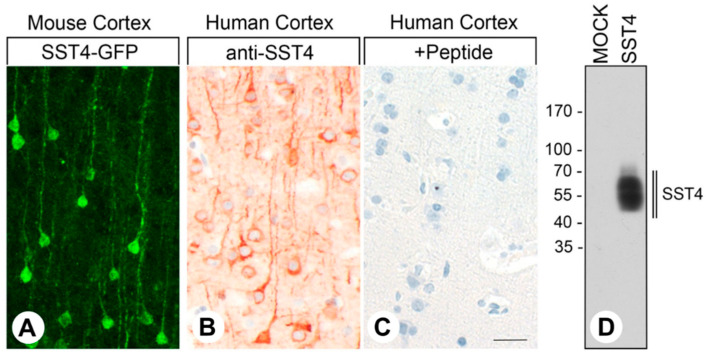
Specificity of the monoclonal rabbit anti-human SST4 antibody 7H49L61. (**A**,**B**) Confocal and light micrographs show the cerebral cortex of SST4-eGFP mice (**A**) and human cortex (**B**,**C**) after anti-GFP immunostaining (**A**) or after immunostaining with the anti-SST4 antibody 7H49L61 (**B**). (**C**) For adsorption controls, 7H49L61 was preincubated with 10 µg/mL of the peptide used for immunization. Scale bar: (**A**) = (**B**) = (**C**) = 50 µm. (**D**) Immunoblot analysis using 7H49L61 antibody for whole-cell preparations from mock- or stably *SST4*-transfected HEK-293 cells. Molecular weight markers (kDa). Representative results of three independent experiments are shown.

The SST4 expression pattern for eGFP immunostaining in the cortex of SST4-GFP knockin mice was virtually identical to the staining pattern for the monoclonal rabbit anti-human SST4 antibody 7H49L61 in human cortex samples with clear staining of the cell bodies and the axons of the cortical pyramidal cells (Figure 4A,B). Pre-incubation of 7H49L61 with its immunizing peptide resulted in a complete extinction of the immunostaining (Figure 4C, +Peptide). In immunoblots of membrane preparations from *SST4*-transfected HEK-293 cells, the antibody recognized a broad band migrating at 50–60 kDa, the expected molecular weight of the glycosylated receptor [15]. In mock-transfected HEK-293 cells, in contrast, no immunosignal could be observed (Figure 4D).

### 2.3. SST4 Expression in Normal Human Tissues

**Figure 5:**
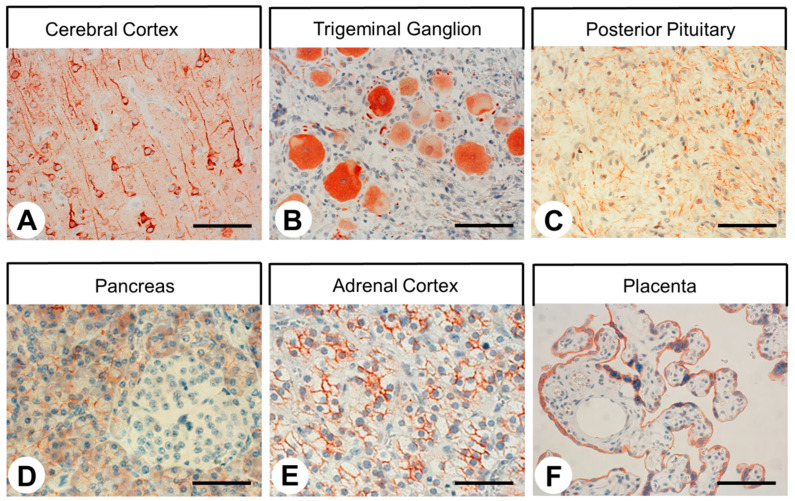
SST4 expression in different normal human tissues. SST4 was detected immunohistochemically in sections of human tissues using antibody 7H49L61. Scale bar: (**A**–**C**,**F**) = 100 µm; (**D**,**E**) = 60 µm.

The rabbit monoclonal anti-SST4 antibody 7H49L61 was then applied in immunohistochemical staining of various human normal tissues. In most cases, immunostaining was localized to the plasma membrane of the cells, but cytoplasmic staining was also observed. As depicted in Figure 5, very strong immunostaining of SST4 was observed in the pyramidal cells of the cortex, in neurons as well as in some satellite cells of the trigeminal ganglia, in nerve fibers within the posterior pituitary gland, in the acinar cells and in the excretory ducts of the exocrine pancreas, in all three layers of the adrenal cortex, and in syncytiotrophoblasts of the placenta. SST4 was also present in the intestinal ganglia, fundic glands of the stomach, Brunner’s glands, epithelium and Paneth cells of the duodenum, bile duct epithelial cells of the liver, and distal and (to a significantly smaller extent) proximal tubules as well as in the glomerular mesangial cells of the kidney. No noticeable staining was observed in the anterior pituitary gland, lung, thyroid gland, liver hepatocytes, pancreatic islet cells, adrenal medulla, spleen, lymph nodes, or bone marrow.

### 2.4. SST4 Expression in Human Tumors

**Figure 6:**
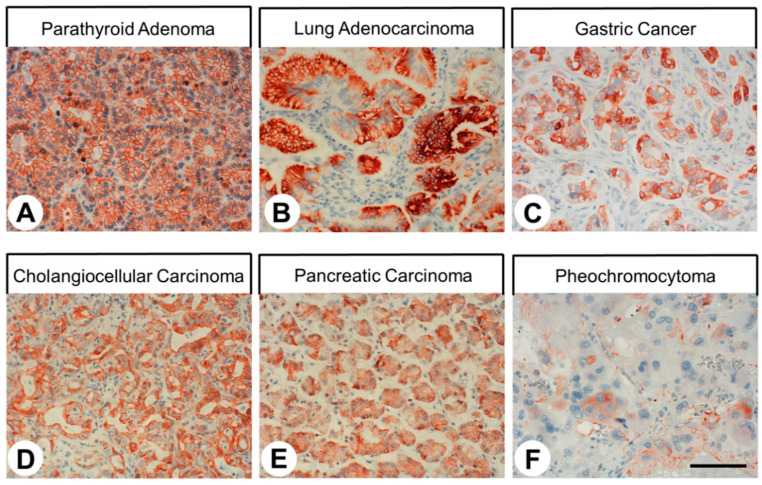
SST4 expression in different neoplastic human tissues. SST4 was detected immunohistochemically in sections of tumor tissues using antibody 7H49L61. Scale bar: (**A**–**F**) = 100 µm.

The patterns of SST4 expression in human tumor samples are summarized in Table 1. Representative examples of immunostaining are shown in Figure 6. Again, both membranous and cytoplasmic staining of cells was observed. Notably, SST4 expression in the tumor samples displayed substantial inter- and intra-individual variability. Pronounced SST4 expression associated with a high number of SST4-positive samples (immunoreactivity score (IRS) ≥ 3) and higher IRS values was seen in glioblastomas, parathyroid adenomas, gastric cancer, pancreatic adenocarcinomas, pheochromocytomas, and lymphomas. However, for most other tumor entities, single samples with high levels of SST4 expression were noted. Interestingly, no noticeable immunostaining was found in small cell lung cancer tissues.

## 3. Discussion

### 3.1. SST4-eGFP Expression in the Knockin Mouse Line

To enable visualization of SST4 expression at the protein level in mice, we generated a SST4-eGFP knockin mouse line. In the present study, we provide strong evidence that the immunosignals obtained with this mouse line indeed reflect SST4 receptor expression. First, the mRNA expression data obtained by in situ hybridization experiments in wild-type mice correlated well with SST4-eGFP protein expression in the knockin mice. Second, with the novel monoclonal SST4 antibody 7H49L61 virtually the same staining pattern was observed in humans, although there may be some species differences, especially with respect to the pituitary and the pancreas, which need to be further evaluated. Third, both staining patterns correspond well to the published mRNA and protein expression data for mice, rats, and humans [15,26,27,28,29,31,34]. Given that monoclonal anti-mouse SST4 antibodies are currently not available, the SST4-eGFP knockin mouse line can serve as a useful tool for the detection of SST4 in mice at the cellular and subcellular level by direct fluorescence microscopy or immunohistochemical techniques using anti-GFP antibodies.

### 3.2. Specificity of the Monoclonal Rabbit Anti-human SST4 Antibody 7H49L61

Unlike polyclonal antibodies, monoclonal antibodies are directed against a single epitope, generally leading to greater specificity, and they are available indefinitely in unlimited amounts with consistent quality. Therefore, we developed a monoclonal anti-SST4 antibody suitable for immunoblots in basic research and for immunohistochemical staining of formalin-fixed paraffin-embedded tissues during routine histopathological examinations. In the present study, we demonstrated that the carboxyl-terminal tail of human SST4 can serve as an epitope for generating a rabbit monoclonal antibody that can be effectively used for both applications. We provided strong evidence that the anti-SST4 antibody 7H49L61 specifically detects its target receptor and does not cross-react with other proteins. First, in immunoblots, 7H49L61 specifically detected its cognate receptor in crude extracts from SST4-transfected HEK-293 cells but not in extracts from mock-transfected cells. Second, 7H49L61 yielded highly efficient staining of distinct cell populations known to express SST4 [31,32,34] in formalin-fixed paraffin-embedded human tissue samples. Third, the SST4-immunosignals were completely abolished after pre-adsorption of the antibody with its immunizing peptide. Finally, virtually identical staining patterns were observed in the human cortical and trigeminal ganglion samples stained with 7H49L61 and in the corresponding tissues of the SST4-eGFP knockin mice. Most notably, however, 7H49L61 is the first SST4 antibody that facilitates detection of bona fide plasma membrane receptors in human formalin-fixed paraffin-embedded tissues.

### 3.3. SST4 Expression in Normal Human Tissues

In the present investigation, a distinct immunostaining for SST4 was found in the cell bodies and the axons of cortical pyramidal cells, consistent with published data obtained using polyclonal antibodies in humans [31] and in rats [15,27,28] as well as in SST4 humanized mice [34]. SST4 has also been identified in neurons and satellite cells of trigeminal and dorsal root ganglia of mice and rats, respectively, by mRNA analysis or with polyclonal antibodies, and it was proposed that the analgesic effects of SST4 agonists are partially mediated by these receptors [22,23,29]. We also found SST4 expression in the intramural ganglia of the gastrointestinal tract, indicating broad expression of SST4 in the peripheral nervous system. Similar to published data both at the mRNA and at the protein level [32,56], no SST4 expression was observed in the anterior pituitary; hence, it is believed that SST4 agonists may represent good analgesics without exerting any major endocrine side effects [20,23]. In contrast, a strong staining of nerve fibers within the posterior pituitary was detected, which has not been reported previously and suggests a possible involvement of SST4 in the regulation of oxytocin and adiuretin secretion from these fibers. While pancreatic islet cells were SST4 negative, in the present study, a distinct staining of acinar cells of the exocrine pancreas was observed. Similar observations have been made in the literature using polyclonal antibodies [32,33] and an involvement of SST4 in the well-known inhibitory role of somatostatin on pancreatic juice excretion but not in insulin or glucagon release was suggested. As reported previously [32], the presence of SST4 was also noted in the glands of the gastric fundus and in the epithelium, Brunner’s glands, and Paneth cells of the duodenum. At these sites, SST4 (and other SSTs) may mediate the inhibitory effects of somatostatin on gastric acid and duodenal bicarbonate secretion [57,58]. The additional presence of SST4 in bile duct epithelia suggests that it also participates in the reduction of bile production by somatostatin [59,60,61]. Consistent with previous reports [32,62], SST4 expression was further observed in the distal tubules and, to a lesser extent, in the proximal tubules and the glomerular mesangial cells of the kidneys. Here, SST4 may be involved in the effects of somatostatin on glomerular vascular tone and on water and electrolyte transport [63]. While SST4 could not be detected in adrenal medulla, in the current study, strong SST4 expression was noticed in all three layers of the adrenal cortex. This finding, which has not been reported before, suggests an involvement of SST4 in the regulation of adrenal cortex hormone secretion. Finally, confirming mRNA expression data [64], in the present investigation, strong SST4 expression was noted in the syncytiotrophoblasts of the human placenta. This suggests a regulatory effect of SST4 on the release of hormones like progesterone, human chorionic gonadotropin, human placental lactogen, or leptin by these cells.

All in all, our studies revealed a unique expression profile and function for SST4 compared to the other SSTs in humans. SST4, unlike the other SSTs, is not expressed in endocrine or neuroendocrine cells and tissues, such as the anterior pituitary, islet cells of the pancreas, neuroendocrine cells of the bronchopulmonary or gastrointestinal tract, or in the adrenal medulla.

### 3.4. SST4 Expression in Human Tumors

For the 26 different tumor entities evaluated for SST4 expression, high expression was noted in glioblastomas, parathyroid adenomas, gastric and pancreatic adenocarcinomas, pheochromocytomas, and lymphomas, but also in the other entities, single cases with high IRS values were observed. In most cases, our results confirmed previous data obtained with polyclonal antibodies on the frequency and intensity of expression in tumors. These entities were papillary and follicular thyroid carcinomas [41,42,43], parathyroid adenomas [53], squamous cell carcinomas of the lung [51], small cell lung cancer [44,48], hepatocellular and cholangiocellular carcinomas [50], urinary bladder cancer [55], prostate adenocarcinomas [37], breast cancer [45,47,54], endometrial and cervical cancer [40], and ovarian cancer [38]. In some instances, previously published expression levels were lower than found in the present investigation, which may be due to the higher sensitivity and specificity of the novel monoclonal antibody 7H49L61. These included medullary thyroid carcinomas [36], adenocarcinomas of the lung [51], gastrointestinal stromal tumors [46], pancreatic adenocarcinomas [52], pheochromocytomas [39], and lymphomas [35,49]. Among the 26 tumor entities that were investigated in the present study, 7 tumor types (glioblastomas, anaplastic thyroid carcinomas, gastric cancer, colon cancer, renal clear cell carcinomas, testicular cancer, and malignant melanomas) were evaluated for SST4 expression at the protein level for the first time.

Overall, SST4 shows a completely different expression pattern compared to the other SST family members in human tumors. While the other SSTs are predominantly found in tumors of (neuro)endocrine origin, SST4 is more prominently present in adenocarcinomas, probably because the SST4 (but not the other SSTs) is already present in the respective normal tissues. Especially in the tumor types with a large number of SST4-positive samples and with high expression intensities in the individual tumors, SST4 may be of diagnostic or therapeutic value. Particularly for glioblastomas, gastric cancer, or pancreatic adenocarcinomas, all with very low 5-year survival rates, new diagnostic or therapeutic options are urgently needed. In these tumor entities, further investigations with larger sample numbers and taking into account the clinical data of the patients are warranted.

## 4. Materials and Methods

### 4.1. Monoclonal Antibody

A rabbit monoclonal antibody (7H49L61) that recognizes the carboxyl-terminal tail of human SST4 was generated in collaboration with and obtained from Thermo Fisher Scientific (Waltham, MA, USA). The peptide used for immunizations of the rabbits was CQQEALQPEPGRKRIPLTRTTTF, corresponding to residues 366–388 of human SST4. This sequence is unique to human SST4; therefore, 7H49L61 does not cross-react with rat or mouse SST4.

### 4.2. Immunoblot Analysis

Human embryonic kidney 293 cells (HEK-293; DMSZ, Braunschweig, Germany), either mock-transfected or transfected with human *Sst4*, were seeded onto poly-L-lysine-coated 60-mm dishes, and grown to 80% confluency. Cells were lysed in detergent buffer [20 mM HEPES (pH 7.4), 150 mM NaCl, 5 mM EDTA, 1% Triton X-100, 10% glycerol, 0.1% SDS, 0.2 mM phenylmethylsulfonylfluoride, 10 mg/mL leupeptin, 1 mg/mL pepstatin A, 1 mg/mL aprotinin, and 10 mg/mL bacitracin]. SST4 was enriched using wheat germ lectin agarose beads. Proteins were separated on a 7.5% SDS-polyacrylamide gel by electrophoresis and immunoblotted onto polyvinylidene fluoride (PVDF) membranes. Blots were incubated with the rabbit monoclonal anti-SST4 antibody 7H49L61 (1:500) followed by a peroxidase-conjugated secondary anti-rabbit antibody (1:5000) (Santa Cruz Biotechnology, Dallas, TX, USA) and detected using enhanced chemiluminescence (Amersham, Braunschweig, Germany).

### 4.3. SST4-eGFP Knockin Mice

For the examination of SST4 expression in mice, a SST4-eGFP knockin mouse line was developed in a C57BL/6 background (Cyagen, Santa Clara, CA, USA). In these mice, the SST4 receptor is expressed as a fusion protein with a green fluorescent protein (eGFP) attached to its carboxyl terminus. Animals were housed in plastic cages under standardized conditions (light-dark cycle 12/12 h, temperature 22 ± 2 °C, humidity 50 ± 10%, pellet diet Altromin 1316 (Altromin, Lage, Germany), water ad libitum). The principles of good laboratory animal care and the German Law on the Protection of Animals, as well as Directive 2010/63/EU, were followed.

### 4.4. Immunohistochemistry on SST4-eGFP Knockin Mouse Tissues

SST4-eGFP knockin mice as well as wild-type littermates of either sex were sacrificed using 5% isoflurane and transcardially perfused with PBS followed by 4% formaldehyde/PBS (pH 7.4). For immunofluorescence stainings, brain and trigeminal ganglia were rapidly dissected and post-fixed in the same fixative for 24 h at room temperature. After immersion in 30% sucrose in TPBS (10 mmol/L Tris, 10 mmol/L phosphate, 155 mmol/L NaCl, pH 7.4) for 48 h at 4 °C, 40-µm cryosections were prepared and subjected to free-floating immunohistochemistry as described previously [65]. Briefly, free-floating sections were successively incubated with 50% methanol in TPBS and with 5% BSA/0.3% Triton X-100 in TPBS before applying the rabbit anti-GFP antibody (ab290, Abcam, Cambridge, UK; 1:7500) at 4 °C overnight. For amplification, the biotin/tyramine method was used: samples were incubated for 2 h with the biotinylated goat anti-rabbit antibody (Dianova, Hamburg, Germany; 1:400) before applying ABC Elite Kit peroxidase (PK-6100, Vector Laboratories, Burlingame, CA, USA) and working buffer containing 0.015% H_2_O_2_ and 7.5 nmol/L biotinylated tyramine. Streptavidin-coupled Cy3 (S11223, Invitrogen, Waltham, MA, USA) was used at 1:500 in working buffer for detection. For double-labeling immunofluorescence experiments, samples were incubated additionally with monoclonal mouse anti-NeuN antibody (MAB377, Millipore, Burlington, MA, USA; 1:300) followed by donkey anti-mouse Cy5 (Dianova, Hamburg, Germany; 1:500).

Specimens were analyzed using an LSM Meta 510 confocal microscope (Carl Zeiss, Jena, Germany).

For immunohistochemical stainings on paraffin sections, organs were quickly removed, fixed for 72 h in 4% formaldehyde/PBS (pH 7.4), dehydrated, and embedded in paraffin blocks. The further procedure was as described below for the human tissue samples. As primary antibody, a rabbit anti-GFP antibody (ab290, Abcam, Cambridge, UK; 1:1000) was used.

### 4.5. In Situ Hybridization Experiments

In situ hybridization was carried out on brain and trigeminal ganglion tissues from C57BL/6 wild-type mice as described previously [66,67] using ^35^S-labeled riboprobes. The *Sstr4* probe corresponded to nucleotides 262–1790 of the coding sequence of the receptor gene and was controlled using the sense strand probe.

### 4.6. Human Tissue Samples

For the evaluation of SST4 expression in different human tumors, 230 formalin-fixed and paraffin-embedded tumor samples (Table 1) were obtained from the Department of Pathology of the University Hospital, Otto-von-Guericke-University, Magdeburg, Germany. Many of the tumor specimens contained adjacent non-malignant tissue that was also analyzed. Tumor-free human tissue samples from the cortex, trigeminal ganglia, pituitary, lung, heart, liver, stomach, duodenum, colon, pancreas, kidneys, adrenals, spleen, lymph nodes, placenta, and bone marrow (n = 3–6 each), obtained from the Department of Pathology of the University Hospital, Otto-von-Guericke-University, Magdeburg, Germany, were also evaluated. Staining patterns were compared to the non-malignant tissues surrounding the tumors.

### 4.7. Immunohistochemistry on Human Tissue Samples

From the paraffin blocks, 4-µm sections were prepared and floated onto positively charged slides. Immunostaining was performed by an indirect peroxidase labeling method as described previously [68]. Briefly, sections were dewaxed, microwaved in 10 mM citric acid (pH 6.0) for 16 min at 600 W, and incubated with 7H49L61 (1:500) overnight at 4 °C. The primary antibody was detected using biotinylated anti-rabbit IgG followed by incubation with peroxidase-conjugated avidin (Vector ABC “Elite” kit; Vector Laboratories, Burlingame, CA, USA). The binding of the primary antibody was visualized using 3-amino-9-ethyl carbazole in acetate buffer (BioGenex, San Ramon, CA, USA). Sections were rinsed, counterstained with Mayer’s hematoxylin, and mounted in Vectamount™ mounting medium (Vector Laboratories, Burlingame, CA, USA). For immunohistochemical controls, 7H49L61 was either omitted or adsorbed for 2 h at room temperature with 10 µg/mL of the peptide used for immunizations.

The staining of SST4 in the tumors was scored with the semiquantitative immunoreactivity score (IRS) according to Remmele and Stegner (1987) [69]. The percentage of positive tumor cells categorized in five grades (no positive cells [0], <10% positive cells [1], 10–50% positive cells [2], 51–80% positive cells [3], and >80% positive cells [4]) was multiplied by the staining intensity categorized in four grades (no staining [0], mild staining [1], moderate staining [2], and strong staining [3]). Thus, IRS values ranging from 0 to 12 were obtained. Only tumor samples with an IRS value ≥3 were considered SST4 positive.

## 5. Conclusions

We developed an SST4-eGFP knockin mouse line that enables SST4 localization in mice in great detail either by direct imaging of the receptor under a fluorescent microscope or by immunohistochemistry using commercially available anti-GFP antibodies. We also generated a rabbit monoclonal anti-human SST4 antibody, 7H49L61, that is well suited for immunoblots in basic research and for visualizing human SST4 in formalin-fixed paraffin-embedded tissues during routine histopathological examinations. Notably, 7H49L61 is the first antibody that detects membrane-bound SST4 receptors in human tissues. Using 7H49L61 enabled us to generate the first unequivocal SST4 expression profile in a wide variety of normal and neoplastic human tissues.

## Figures and Tables

**Table 1:** Presence of SST4 in different human tumor samples. Mean IRS values ≥ 3.0 are marked in bold. Min: minimum values; Max: maximum values.

Tumor Type (Total Number of Samples)	SST4 Positive Tumors (n)	Immunoreactivity Score (IRS)		
		Mean	Min	Max
Glioblastoma (9)	9	**6.78**	4.5	9
Thyroid carcinoma (36)	18	2,95	0	9
- papillary (11)	5	2.05	0	5
- follicular (10)	3	2.30	0	7.5
- medullary (7)	6	**5.18**	0	9
- anaplastic (8)	4	**3.06**	0	8
Parathyroid adenoma (10)	10	**9.35**	3	12
Lung cancer (31)	10	2,19	0	9
- Adenocarcinoma (12)	9	**4.50**	0	9
- Squamous cell carcinoma (10)	0	0.68	0	2
- Small cell lung cancer (9)	1	0.78	0	5
Gastric cancer (9)	9	**8.06**	4.5	10
Colon carcinoma (9)	6	**3.70**	1	9
Gastrointestinal stromal tumor (10)	8	**5.03**	0	10
Hepatocellular carcinoma (11)	3	1.55	0	6
Cholangiocellular carcinoma (9)	3	**3.70**	0	12
Pancreatic adenocarcinoma (11)	10	**6.27**	2	9
Renal clear cell carcinoma (8)	0	0.63	0	2
Pheochromocytoma (7)	7	**6.50**	4	10.5
Bladder Cancer (7)	6	**4.68**	2	7.5
Prostate adenocarcinoma (12)	5	2.20	0	7.5
Testicular cancer (12)	8	**3.60**	0	7.5
Breast carcinoma (9)	6	**3.47**	0	6.25
Endometrial cancer (10)	2	1.53	0	4.5
Cervical cancer (9)	3	1.40	0	4.5
Ovarian cancer (10)	4	2.44	0	5
Lymphoma (12)	10	**6.02**	1.5	10
Melanoma (5)	3	**3.20**	1	6

## Data Availability

The data that support the findings of this study are all contained within the article.

## Abbreviations

eGFP	enhanced green fluorescent protein
IRS	immunoreactivity score
SST	somatostatin receptor
